# Supplementary material for: The interactome of intact mitochondria by cross-linking mass spectrometry provides evidence for coexisting respiratory supercomplexes
Source: Mol Cell Proteomics. 2017 Dec 8;17(2):216–32. doi: 10.1074/mcp.RA117.000470 (PMC5795388; doi:10.1074/mcp.RA117.000470)
Supplement: Supplemental Data [file supp_RA117.000470_133922_0_supp_23978_fzffwf.zip › spectra_annotation/mito_DR_spectra_annotation/243-1-5-1-1-1.pdf]

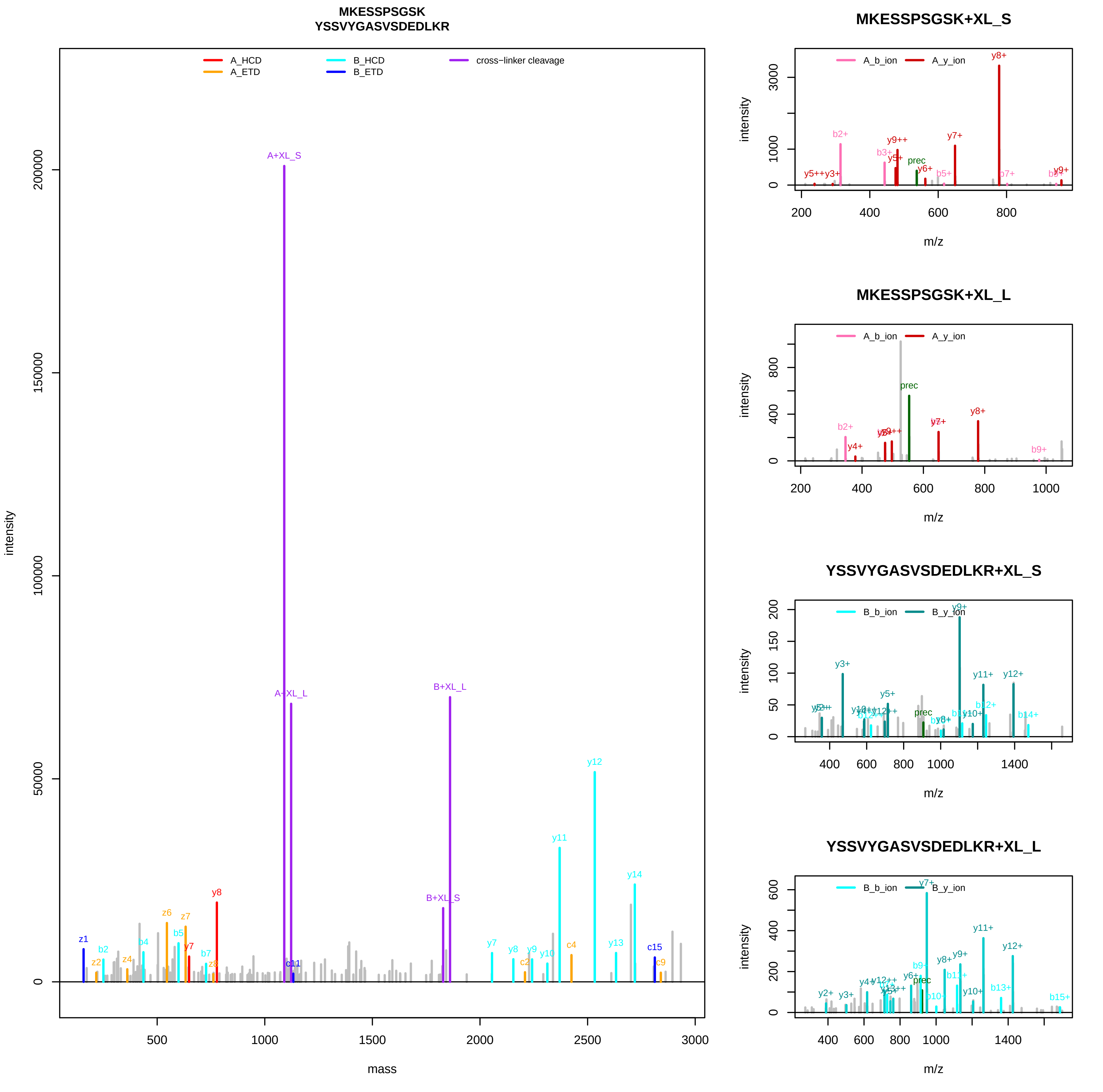

MKESSPSGSK
YSSVYGASVSDEDLKR
A_HCD
A_ETD
B_HCD
B_ETD
cross−linker cleavage
A+XL_S
A+XL_L
B+XL_L
B+XL_S
y12
y11
y14
y13
y8
y7
z1
b2
z2
b4
z4
z6
z7
b5
y7
b7
z8
c11
y9
y10
c2
c4
c15
c9
intensity
mass
0
50000
100000
150000
200000
500
1000
1500
2000
2500
3000
MKESSPSGSK+XL_S
A_b_ion
A_y_ion
y8+
b2+
y9++
y7+
b3+
y5+
prec
y6+
y5++
y3+
b5+
b7+
b9+
y9+
m/z
MKESSPSGSK+XL_L
prec
y8+
y7+
b2+
y9++
y4+
b9+
YSSVYGASVSDEDLKR+XL_S
B_b_ion
B_y_ion
y9+
y3+
y11+
y12+
y5+
b12+
prec
y10+
b14+
YSSVYGASVSDEDLKR+XL_L
y7+
y11+
y12+
y9+
y8+
b9+
y6+
prec
b11+
y4+
y2+
y3+
y10+
b13+
b10+
b15+
